# Association between diurnal core body temperature rhythm and diurnal variation in blood biomarkers of dementia

**DOI:** 10.1002/alz70856_106849

**Published:** 2026-01-08

**Authors:** Rodrigo Triana‐Del Rio, Emily Blank, Anushka Thummalapenta, Daphne Valencia, Aryan Govil, David M Rapoport, Indu Ayappa, Andrew Varga, Ankit Parekh, Ricardo S. Osorio, Esther M Blessing

**Affiliations:** ^1^ NYU Langone School of Medicine, NYC, NY, USA; ^2^ NYU Langone Hospital, New York City, NY, USA; ^3^ Icahn Mount Sinai School of Medicine, NYC, NY, USA; ^4^ NYU Langone Hospital, NYC, NY, USA; ^5^ Icahn School of Medicine at Mount Sinai, New York, NY, USA; ^6^ Icahn School of Medicine, New York, NY, USA; ^7^ Center for Sleep and Brain Health, Department of Psychiatry, NYU Langone Health, New York, NY, USA; ^8^ New York University, New York City, NY, USA

## Abstract

**Background:**

Core CSF and plasma biomarkers of Alzheimer's Disease (AD) Aβ40, Aβ40, total tau and Ptau‐217 exhibit a diurnal pattern, being higher in the evening vs morning in most, but not all reports. The mechanisms underlying this pattern remain unknown. We recently showed that diurnal∆ (7PM ‐ 7AM levels) in plasma total tau levels in older adults correlated with core body temperature (Tc) diurnal change (Tdrop), consistent with temperature‐dependent tau secretion shown in vivo and in vitro (Canet et al., 2025, JCI, in press). Here, we extend this analysis to plasma Aβ40, Aβ42, and Ptau‐217 levels, and examine sex differences.

**Method:**

We recorded Tc in 44 older adults aged 65±5.24 years (40 cognitively normal, 4 MCI) with ingestible telemetry during 48 hours including two nights in the sleep lab. Blood was sampled at 7 pm and 7 am (four occasions total), creating three plasma diurnal∆ outcomes. Tdrop (Tc during wake ‐ T during sleep on night 2) was calculated for multiple clock intervals (3 hours either side of Tmax and Tmin) to explore correlations with diurnal∆ for target plasma levels quantified with SIMOA quanterix assays.

**Result:**

Females (*n* = 25) vs males (*n* = 19) showed higher Tc (by ∼0.25°C) from 6 am – 6 pm, and a larger Tdrop, *p* < 0.05. Tdrop was significantly correlated with diurnal∆ for all four biomarkers and for all three diurnal∆ outcomes for multiple (>10) clock intervals as follows: Aβ42 (r=0.64–0.82, *n* = 17), Aβ40 (r=0.59–0.73, *n* = 17), Aβ42/Aβ40 ratio (r=0.65–0.83, *n* = 17), and pTau‐217 (0.54–0.92, *n* = 11, with the exception of plasma diurnal∆3, *p* > 0.05). While PM levels were not significantly higher compared to AM levels for Aβ40, Aβ42 and Ptau217, a median split by Tdrop showed that this was the case in the larger Tdrop group, *p* < 0.05 all comparisons. Females vs males tended to have larger plasma diurnal∆, which was significant for diurnal∆2 for Aβ42, tau and *p*‐tau217, *p* < 0.05, all comparisons.

**Conclusion:**

Core body temperature (Tc) diurnal change correlates with that in core plasma biomarkers of AD and may be a mechanism underlying this diurnal pattern. Measuring Tc will inform variability between sexes, subjects and study outcomes.

